# Does the extension of the type of hysterectomy contribute to the local control of endometrial cancer?

**DOI:** 10.1007/s10147-019-01458-2

**Published:** 2019-05-08

**Authors:** Tetsuya Hasegawa, Megumi Furugori, Kazumi Kubota, Mikiko Asai-Sato, Aiko Yashiro-Kawano, Hisamori Kato, Yuka Oi, Hiroyuki Shigeta, Keiko Segawa, Masakazu Kitagawa, Yuko Mine, Haruya Saji, Reiko Numazaki, Yasuyo Maruyama, Emi Ohnuma, Hanako Taniguchi, Ken Sugiura, Etsuko Miyagi, Tatsuya Matsunaga, Yokohama City University Gynecologic Oncology Research Group (YCUGO-ReG)

**Affiliations:** 1 Yamato Municipal Hospital, 8-3-6 Fukaminishi, Yamato, Kanagawa 242-8602 Japan; 2 Department of Obstetrics and Gynecology, Yokohama City University School of Medicine, 3-9 Fukuura, Kanazawa-ku, Yokohama, Kanagawa 236-0004 Japan; 3 Kanagawa Cancer Center, 2-3-2 Nakao, Asahi-ku, Yokohama, Kanagawa 241-8515 Japan; 4 Yokohama Municipal Citizen’s Hospital, 56 Okazawa-chou, Hodogaya-ku, Yokohama, Kanagawa 240-8555 Japan; 5 Saiseikai Yokohamashi Nanbu Hospital, 3-2-10 Kounanndai, Kounan-ku, Yokohama, Kanagawa 234-0054 Japan; 6 Yokohama City University Medical Center, 4-57 UraFune-cho, Minami-ku, Yokohama, Kanagawa 232-0024 Japan; 7 Fujisawa City Hospital, 2-6-1 Fujisawa, Fujisawa, Kanagawa 251-8550 Japan; 8 Yokohama Minamikyosai Hospital, 1-21-1 Mutsuurahigashi, Kanazawa-ku, Yokohama, Kanagawa 236-0037 Japan; 9 Odawara Municipal Hospital, 46 Hisano, Odawara, Kanagawa 250-8558 Japan; 10 Yokohama Rosai Hospital, 3211 Kodukue-chou, Kouhoku-ku, Yokohama, Kanagawa 222-0036 Japan; 11 Yokosuka Kyosai Hospital, 1-16 Yonegahamadouri, Yokosuka, Kanagawa 238-8558 Japan

**Keywords:** Endometrial cancer, Local recurrence, Type of hysterectomy

## Abstract

**Objective:**

To examine the necessity and sufficiency of different types of hysterectomy for the surgical treatment of endometrial cancer.

**Methods:**

This was a multicenter collaborative study conducted by 11 institutions. Among patients with stage I–III endometrial cancer who underwent surgery as the initial treatment (only chemotherapy was provided if adjuvant therapy was needed) from 2001 to 2012, we retrospectively examined the type of hysterectomy, clinicopathological factors, recurrence rate over a maximum period of 5 years, and the site of recurrence. The local recurrence rate was examined by univariate and multivariate analyses.

**Results:**

Among 1335 patients, 982 (73.6%) underwent simple hysterectomy (SH) and 353 (26.4%) underwent modified radical hysterectomy (mRH) and were observed for a mean duration of 51.8 months. No significant difference was observed in the rate of local recurrence between the SH and mRH groups (*p* = 0.928). In multivariate analysis, clinicopathological factors independently associated with localized recurrence included postmenopausal status [hazard ratio (HR) 5.036, 95% confidence interval (CI) 1.506–16.841, *p* = 0.009], with stages II (HR 3.337, 95% CI 1.701–6.547, *p* < 0.001) and III (HR 2.445, 95% CI 1.280–4.668, *p* = 0.007), vs stage I and histological type 2 (HR 1.610, 95% CI 0.938–2.762, *p* = 0.001).

**Conclusions:**

For endometrial cancer patients requiring surgery, the selection of a more extensive type of hysterectomy did not reduce the rate of local recurrence. Therefore, there is little significance in performing mRH in such cases.

## Introduction

During surgery for malignant tumors, the surgeon should ensure that the extent of excision is sufficient to achieve a therapeutic effect while choosing a minimally invasive approach as much as possible to reduce adverse events associated with surgery.

The Japanese guidelines for endometrial cancer 2018 [1] recommend simple hysterectomy for cases with a preoperative diagnosis of stage (grade B), although modified radical hysterectomy (mRH) can also be selected (grade C1). In addition, for a preoperative diagnosis of stage, mRH or radical hysterectomy (RH) is recommended (grade C1). Indeed, in a Japanese questionnaire survey, the selected surgical procedures varied [2]. Among our affiliated institutions, modified radical hysterectomy has been preferentially performed regardless of the disease stage to secure the vaginal wall resection length or ensure the extrafascial approach. Other institutions perform simple hysterectomy or select surgical procedures on a case-by-case basis. The selection of hysterectomy varies widely by institution, which is similar to the nationwide trend in Japan. Systemic chemotherapy is recommended as the postoperative treatment for moderate and high risk cases of endometrial cancer, and pelvic radiation therapy is not recommended as the first choice. In Europe and North America, many cases undergo radiation therapy, which is likely to be more effective for local control than chemotherapy. The differences between hysterectomy methods are rarely reported; however, these should be discussed because chemotherapy is performed in Japan.

For the surgical treatment of endometrial cancer, various types of hysterectomy are performed that differ in terms of the level of invasiveness, rate of complications, duration of operation, presence or absence of blood transfusion, and the degree of urinary disturbance [3]. However, the difference in prognosis according to the type of hysterectomy performed is controversial. Within the Japanese treatment background for endometrial cancer, the most suitable type of hysterectomy for the surgical treatment of endometrial cancer is primarily selected with the aim of maximizing the therapeutic effect and minimizing invasiveness.

In the present study, we examined whether a more suitable type of hysterectomy can be selected by focusing on the rate of local recurrence, which is likely to be directly affected by the type of hysterectomy. The primary endpoint was local recurrence rate, which is thought to reflect differences in the hysterectomy method used, rather than the survival rate or disease-free survival rate, which are greatly affected by pelvic or para-aortic lymphadenectomy, or the quality of these procedures. The purpose of the present study was to examine the necessity and sufficiency of different types of hysterectomy based on the site (i.e., local or distal) and the rate of recurrence of endometrial cancer under the treatment background in Japan.

## Patients and methods

This study included patients with endometrial cancer who underwent surgery as an initial treatment from January 1, 2001 to December 31, 2012 at 11 institutions, including Yokohama City University Medical Center, Kanagawa Cancer Center, Yokohama Municipal Citizen’s Hospital, Saiseikai Yokohamashi Nanbu Hospital, Yokohama City University Medical Center, Fujisawa City Hospital, Yokohama Minamikyosai Hospital, Odawara Municipal Hospital, Yokohama Rosai Hospital, Yokosuka Kyosai Hospital, and Yamato Municipal Hospital. The patients were retrospectively examined on the basis of their medical records with the approval of the ethical review board of each institution.

Because all the surgeons in this study were trained at the same university hospital, common surgical procedures were generally performed.

The study population included patients with endometrial cancer at surgical stages I–III based on the 2008 International Federation of Gynecology and Obstetrics (FIGO) who underwent abdominal simple hysterectomy (SH, type I hysterectomy) or abdominal modified radical hysterectomy (mRH, type II hysterectomy) as the initial treatment [4]. Definitions of hysterectomy performed in facilities that participate in this clinical trial are as follows. SH: after parametrium and the ascending branch of the uterine artery were resected at the level of internal os without isolation of uterine artery and ureter, parametrium was clamped, ligated, and cut in order not to cut into the uterus toward inferior along with cervix of the uterus and vaginal canal. Then, vaginal canal was dissected at the level of fornix of vagina. mRH: after uterine artery was dissected and elevated from the umbilical ligament bifurcation, and ureter was detached from broad ligament, ureter was relocated outside and anterior leaf of vesicouterine ligament was resected. Posterior leaf of vesicouterine ligament and cardinal ligament were clamped and ligated. Then, the vaginal wall was resected approximately 2 cm.

Patients were observed for a maximum of 5 years. Patients who were seen prior to the revised 2008 FIGO staging classification [5] were enrolled after correction for staging. Cases where it was impossible to correct the stage because of a lack of parameters were excluded. At seven institutions, SH was mainly performed and at two institutions, mRH was mainly performed to secure the vaginal wall resection length regardless of the preoperative staging. At the other two institutions, the surgical methods were decided according to the individual case. At these two institutions, mRH was performed when stage II was suspected as a preoperative diagnosis with magnetic resonance imaging (MRI), cervical curettage. Patients with stage IV disease and/or exhibiting residual cancer were excluded because localized recurrence was highly likely, regardless of the prognosis. Patients who received preoperative chemotherapy and/or radiotherapy, those who received adjuvant radiotherapy, and those who exhibited a clear residual tumor at the time of the initial surgery were excluded, because these factors are likely to affect local recurrence. Furthermore, patients who underwent (RH) as the initial surgical treatment were excluded. Because most of the cases in which RH was performed had a preoperative diagnosis of cervical cancer and only four cases in which RH was performed were stage II–III endometrial cancer at preoperative diagnosis, the indication for surgery was unclear. Furthermore, there were too few cases for appropriate statistical analyses.

The examination items included age, body mass index (BMI), menopause, surgical procedure, including the type of hysterectomy and the presence or absence of lymph node dissection, histological type, myometrial invasion, vascular invasion, maximum tumor diameter, postoperative treatment, recurrence site, 5-year progression-free survival rate (PFS) and overall survival rate (OS). Regarding menopause, we compared premenstrual and peri-menopausal periods and postmenopausal states, where amenorrhea lasted for 1 year. Regarding pathological type, type I was classified as endometrioid carcinoma G1, G2 and mucinous carcinoma, and others were classified as type II [6]. The primary endpoints examined were the site of recurrence (local or distal) and associated recurrence rates according to the different types of hysterectomy and the period until recurrence within 5 years. Localized recurrence was defined as recurrence within the vagina and vaginal stump, as well as intrapelvic recurrence other than that in the regional lymph nodes within the pelvis. Recurrence was diagnosed on the basis of histology and/or imaging via computed tomography (CT), MRI, and positron emission tomography–CT (PET–CT).

The parameters of surgical procedures were analyzed using the Mann–Whitney *U* test, Chi-square test, and Mantel–Haenszel test. With the maximum observation period set at 60 months, the survival period until localized recurrence was analyzed using the Kaplan–Meier method, log-rank test, and Cox’s proportional hazard model. Values of *p* < 0.05 were considered significant. Statistical analyses were performed using SPSS software Ver. 20 (IBM Corp., Armonk, NY, USA).

## Results

Data were collected from 1655 patients, among whom 320 were ineligible because of RH (*n* = 23), radiotherapy (*n* = 24), sarcoma (*n* = 17), residual tumor (*n* = 3), other reasons (*n* = 19) or missing data for various important parameters (*n* = 234).

SH and mRH were performed on 982 (73.6%) and 353 (26.4%) patients, respectively. SH was mainly performed at seven institutions: SH was performed on 762 (96.2%) patients, whereas mRH was performed on 30 (3.8%) patients. At two institutions, mRH was mainly performed: SH on 95 (27.9%) patients and mRH on 246 (72.1%) patients. At two other institutions, both procedures were performed at an equal rate: 77 (50%) patients underwent SH or mRH.

Of the 1335 patients who underwent SH and mRH, the mean observation period was 51.8 (0–60) months. The clinicopathological backgrounds for the SH and mRH groups are shown in Table 1. Compared with patients in the SH group, those in the mRH group had a significantly lower BMI (*p* = 0.008), significantly higher rates of pelvic (*p* < 0.001) and para-aortic lymph node dissection (*p* < 0.001), and a significantly smaller maximum tumor diameter (*p* = 0.002). In contrast, no difference was observed in terms of age, age at menopause, postoperative 2008 FIGO stage, myometrial invasion, histological type (1 or 2), the presence or absence of vascular invasion, rate of positive intraperitoneal washing cytology, and rate of postoperative chemotherapy between patients in the two groups.

**Table 1:** Clinical and tumor characteristics according to the surgical procedure

Hysterectomy type	SH (*n* = 982)	MRH (*n* = 353)	Total (*n* = 1335)	*p*
Age, median (range)	59 (26–88)	58 (27–85)	59 (26–88)	0.077
BMI, median (range)	23.1 (13.2–54.1)	22.5 (13.7–42.6)	22.9 (13.2–54.1)	**0.008**
Menopause				
Pre/peri	256 (26.1%)	103 (29.2%)	359 (26.9%)	0.263
Post	726 (73.9%)	250 (70.8%)	976 (73.1%)	
FIGO stage				
I	769 (78.3%)	281 (79.6%)	1050 (78.7%)	0.871
II	75 (7.6%)	26 (7.4%)	101 (7.6%)	
III	138 (14.1%)	46 (13.0%)	184 (13.8%)	
Pelvic lymph node dissection				
Not performed	429 (43.7%)	93 (26.3%)	522 (39.1%)	**< 0.001**
Performed	553 (56.3%)	260 (73.7%)	813 (60.9%)	
Para-aortic lymph node dissection				
Not performed	808 (82.3%)	251 (71.1%)	1059 (79.3%)	**< 0.001**
Performed	174 (17.7%)	102 (28.9%)	276 (20.7%)	
Myometrial invasion				
< 1/2	677 (68.9%)	242 (68.5%)	919 (68.8%)	0.419
≥ 1/2	305 (31.1%)	111 (31.4%)	416 (31.2%)	
Histologic type				
Type 1	756 (77.0%)	271 (76.8%)	1027 (76.9%)	0.941
Type 2	226 (23.0%)	82 (23.2%)	308 (23.1%)	
Vascular invasion				
Negative	722 (73.5%)	247 (70.0%)	969 (72.6%)	0.211
Positive	260 (26.5%)	106 (30.0%)	366 (27.4%)	
Maximum tumor diameter, median (range), mm	40.0 (0–180)	35.0 (0–200)	38 (0–200)	**0.002**
Peritoneal washing cytology				
Negative	789 (80.3%)	291 (82.4%)	1080 (80.9%)	0.117
Positive	193 (19.7%)	52 (17.5%)	255 (19.1%)	
Adjuvant chemotherapy				
Not done	608 (61.9%)	199 (56.4%)	807 (60.4%)	0.076
Done	374 (38.1%)	154 (43.6%)	528 (39.6%)	

Bold numbers indicate statistically significant differences (*p* < 0.05)

*BMI* body mass index, *SH* simple hysterectomy, *mRH* modified radical hysterectomy, *FIGO* International Federation of Gynecology and Obstetrics

PFS and OS with the upper limit of 60 months that were estimated by Kaplan–Meier analysis showed 54.98 and 54.33 months in SH group and mRH group for PFS, respectively, 56.76 and 57.15 months in SH group and mRH group for OS, respectively. *p* values by log-rank test were 0.4553 and 0.7913 for PFS and OS, respectively, which indicated no significant differences.

**Fig. 1:**
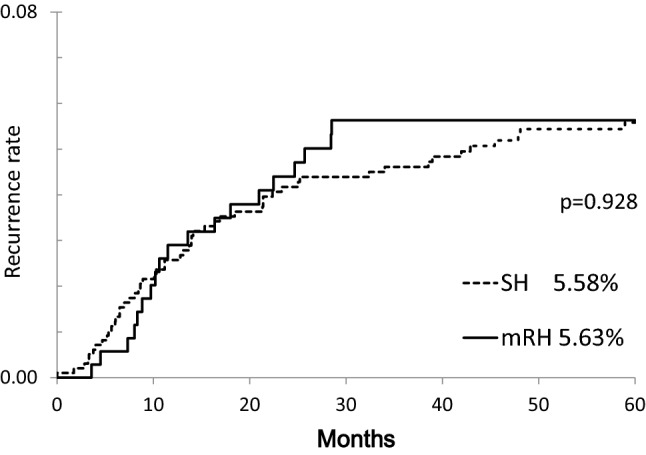
Local recurrence curves according to the surgical procedure

**Fig. 2:**
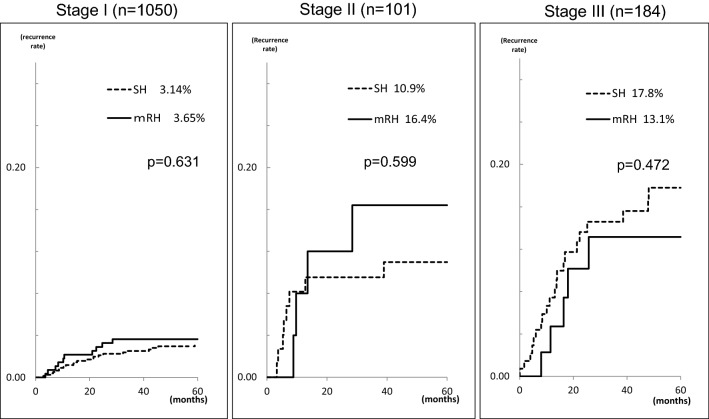
Local recurrence curves by stage according to the surgical procedure

Table 2 shows the number of patients with recurrence and the site of recurrence according to the surgical procedure. Recurrence developed in 116 (11.8%) patients in the SH group and in 47 (13.3%) patients in the mRH group. The number of multiple sites of recurrence was 146 in the SH group and 60 in the mRH group; among these, recurrence was localized in 53 (36.3%) sites in the SH group and in 19 (31.6%) sites in the mRH group. Based on the Kaplan–Meier method, no significant difference was observed in the rates of local recurrence between the SH and mRH groups (Fig. 1). A similar analysis was also conducted for each staging, which revealed no significant difference between groups (Fig. 2). Univariate analysis of other clinicopathological factors revealed a significantly higher rate of localized recurrence for stage I vs stages II and III (*p* < 0.001), age ≥ 62 years (*p* < 0.001), postmenopausal status (*p* < 0.001), histological type 2 (*p* < 0.001), the presence of vascular invasion (*p* < 0.001), maximum tumor diameter ≥ 52 mm (*p* < 0.001), myometrial invasion depth ≥ 1/2 (*p* < 0.001), positive intraperitoneal lavage cytology (*p* < 0.001), and receiving postoperative chemotherapy (*p* < 0.001) (Table 3). Furthermore, according to the receiver operating characteristic (ROC) curve, the cut-off values for localized recurrence were set at 62 years for age and 52 mm for maximum tumor diameter. Multivariate analysis of these factors identified postmenopausal status and stages II and III as significant risk factors for localized recurrence (Table 4). The type of hysterectomy was not a significant risk factor for the rate of local recurrence.

**Table 2:** Site of recurrence according to surgical procedure

	SH (*n* = 982)	mRH (*n* = 353)	Total (*n* = 1335)
No recurrence cases	866 (88.2%)	306 (86.7%)	1172 (87.8%)
Recurrence cases	116 (11.8%)	47 (13.3%)	163 (12.2%)
Number of recurrence sites	146	60	206
Local recurrence	53 (36.3%)	19 (31.6%)	72 (34.9%)
Tumor in the pelvic cavity (except lymph nodes)	28 (19.2%)	6 (10.0%)	34 (16.9%)
Vaginal stump or vagina	28 (17.1%)	13 (21.6%)	38 (18.4%)
Others	93 (63.7%)	41 (68.3%)	134 (65.0%)
Pelvic lymph nodes	19 (13.0%)	5 (8.3%)	24 (11.6%)
Para-aortic lymph nodes	25 (27.1%)	13 (21.6%)	38 (18.4%)
Distant lymph nodes	7 (4.8%)	3 (5.0%)	10 (4.8%)
Distant metastasis	32 (21.9%)	16 (26.6%)	48 (23.3%)
Carcinomatous peritonitis	3 (2.1%)	2 (3.3%)	5 (2.4%)
Bones	4 (2.7%)	1 (1.6%)	5 (2.4%)
Others (intra-abdominal tumor, etc.)	3 (2.1%)	1 (1.6%)	4 (1.9%)

*SH* simple hysterectomy, *mRH* modified radical hysterectomy

*p* = 0.772

**Table 3:** Clinicopathological factors and rate of local recurrence

Characteristics	*p*
Type of hysterectomy, SH vs mRH	0.928
FIGO stage	
I vs II	**< 0.001**
I vs III	**< 0.001**
Age, < 62 years vs ≥ 6 years^a^	**< 0.001**
Menopause	**< 0.001**
BMI, ≥ 22.82 vs < 22.82^a^	0.093
Histologic type, type 1 vs type 2	**< 0.001**
Vascular invasion	**< 0.001**
Maximum tumor diameter, ≤ 52 mm vs > 52 mm^a^	**< 0.001**
Myometrial invasion deeper than 50%	**< 0.001**
Positive peritoneal washing cytology	**< 0.001**
Adjuvant chemotherapy	**< 0.001**
Lymph node dissection	0.527

Bold numbers indicate statistically significant differences (*p* < 0.05)

*FIGO* International Federation of Gynecology and Obstetrics, *BMI* body mass index

^a^Cut-off value calculated from the receiver operating characteristic (ROC) curve

**Table 4:** Correlation between clinicopathological factors and local recurrence

Characteristics	HR	95% CI	*p*
Menopause	**6.791**	**2.120–21.759**	**0.001**
FIGO stage			
I vs II	**3.537**	**1.803–6.939**	**< 0.001**
I vs III	**2.913**	**1.585–5.356**	**0.001**
Histologic type type 1 vs type 2	**3.975**	**2.371–6.666**	**< 0.001**
Vascular invasion	1.652	0.948–2.879	0.077

Bold numbers indicate statistically significant differences (*p* < 0.05)

Multivariate Cox’s regression analysis with step wise method was performed using the following covariates: type of hysterectomy, FIGO stage, pre- or postmenopausal status, histological type 2, vascular invasion, tumor diameter ≥ 52 mm, myometrial invasion depth ≥ 1/2, and positive intraperitoneal lavage cytology

*FIGO* International Federation of Gynecology and Obstetrics, *HR* hazard ratio, *CI* confidence interval

## Discussion

The results of the present study suggest that for the initial surgical treatment of endometrial cancer, extending the type of hysterectomy from SH to mRH did not reduce the rate of local recurrence. Furthermore, we found that postmenopausal status and surgical stages II and III were factors that affected the rate of local recurrence.

The present study examined the effects of different types of hysterectomy on the recurrence and prognosis of endometrial cancer, with a focus on the rate of local recurrence, which was defined as intrapelvic, other than recurrence of the lymph nodes. Most previous studies [7–10] investigating the local recurrence of endometrial cancer described adjuvant radiotherapy. Because radiotherapy has a major localized therapeutic effect, local recurrence cannot be considered to purely reflect the differences in surgical procedures. In the present study, patients who received adjuvant radiotherapy were excluded; thus, differences between the types of hysterectomy only reflected the rate of localized recurrence. Because the control of local recurrence greatly influences progression-free survival (PFS) and overall survival (OS), we believe that the results of the present study are of significance.

The present study included a large subject sample of 1355 patients with stages I–III endometrial cancer who underwent surgery as the initial treatment. Furthermore, the study had a sufficiently long observation period (maximum observation period, 60 months and mean observation period, 51.8 months, which was comparable with past reports). However, there were several limitations in this study as follows: (1) it was a retrospective study; (2) it was a multicenter study including a large number of institutions (*n* = 11) with non-identical surgical procedures between institutions; (3) selection criteria to choose the surgical method were unclear; (4) Surgical procedures may be different among hospitals; (5) postoperative therapy was chemotherapy only; and (6) there were some differences in patient background between the two procedures: patients in the mRH group had significantly higher rates of pelvic lymph node dissection (*p* < 0.001) and para-aortic lymph node dissection (*p* < 0.001), significantly lower BMI (*p* = 0.008), and significantly smaller maximum tumor diameter (*p* = 0.002) compared with those in the SH group. However, we believe our results are still valuable. The differences in surgical procedures among hospitals were minimal and the selection criteria were similar because the personnel involved in the study underwent similar training. Regarding patient background, we excluded recurrence in the regional lymph nodes within the pelvis. There were no differences in surgical stage, myometrial invasion, histological type (type 1 or type 2), and vascular invasion between the two groups. Furthermore, this study was conducted to determine whether mRH exceeded SH; however; BMI and tumor size were more prevalent factors for mRH. Therefore, we consider that these variables had a minimal impact on the results, which did not show the superiority of mRH compared with SH.

The types of hysterectomy for endometrial cancer include SH and mRH, which differ in terms of the length of vaginal wall and parametrial tissue resection. Two previous studies [10, 11] described a relationship between vaginal wall resection and the local recurrence of endometrial cancer. However, no studies have provided high-level evidence for this relationship. In one study [10], the vaginal wall and parametric tissue were also severely resected in the SH operation. The other study [11] was retrospective that only included a small number of cases. In this study, vaginal wall resection length was initially considered an item to be examined, but statistical analysis was not possible because of high data loss.

A retrospective study conducted in Japan by Morikazu et al. on SH and mRH for 247 patients with stage I and stage II endometrial cancer [12] reported the PFS and OS did not significantly differ between the groups. In a randomized controlled trial of extrafascial SH and mRH performed on 520 patients with stage I endometrial cancer, Mauro et al. reported no significant difference between the two groups in terms of disease-free survival and OS [10]. Taek et al. examined 133 patients with cervical invasion equivalent to stage II endometrial cancer [13] and concluded that mRH and RH were not effective. Furthermore, a study of 819 patients with type 1 stage II endometrial cancer, RH performed on 273 patients and SH performed on 546 patients from the Surveillance, Epidemiology, and End Results database [14] reported that RH did not improve the survival rate. These results are consistent with our study targeting cases with stage I–III endometrial cancer, with local recurrence as the primary endpoint.

In contrast, a retrospective study for stage I endometrial cancer conducted on 340 patients [11] reported that those in whom 15 mm of the vaginal wall was resected had a longer PFS and OS compared with patients who did not undergo vaginal wall resection. However, this study did not specify the indications for lymph node dissection, radiotherapy, and chemotherapy in the comparison of PFS and OS; thus, other factors might need to be taken into consideration when interpreting these results. Together with our results, it is highly likely that PFS and OS are not solely affected by the type of hysterectomy but are largely affected by the presence or absence of lymph node dissection, as well as the selection and details of adjuvant treatment.

In the present study of stage III cases with no evidence of residual tumor, there was no difference in the local recurrence rate by difference in hysterectomy methods. However, in stage IIIb cases with parametrial invasion or tumor extending into vaginal wall, there is a high possibility that surgical resection margins may be inadequate by total hysterectomy or modified radical hysterectomy. For patients with stage III endometrial cancer, it is currently unknown whether PFS and OS are affected by the type of hysterectomy because we were unable to find any study on the types of hysterectomy in this stage.

A phase III trial in Japan conducted on postoperative pelvic radiotherapy versus cyclophosphamide–doxorubicin–cisplatin (CAP) chemotherapy for patients with intermediate-to-high risk cancer, with 193 patients per treatment group, showed no significant differences in PFS and OS [15]. Because postoperative chemotherapy is thought to have equivalent effects to pelvic radiotherapy, we mainly provided chemotherapy for patients who had high recurrence risk after surgery.

In the present study, factors associated with local recurrence included stage, postmenopausal status, and histological type 2. Previous studies reported that factors related to PFS included age, tumor grade, myometrial invasion, and stage [10–12] and those affecting OS included age, tumor grade, stage, and vascular invasion [1, 11, 12, 16]. These results are similar to those of the present study. In particular, postmenopausal state was extracted as a risk factor in this study. Although the stage distribution remained the same in postmenopausal group compared with the pre-menopausal or peri-menopausal groups, there were more type 2 cases with deeper myometrial invasion and more positive vascular invasion. This was likely to have been extracted as a risk factor because reduction treatment may be selected at a high rate, similar to older age. Therefore, the factors involved in PFS and OS can be considered comparable with those involved in local recurrence observed in the present study. Thus, controlling the rate of local recurrence may help to greatly improve the PFS and OS of endometrial cancer patients.

Currently, laparoscopic and robot-assisted surgeries are gaining popularity worldwide, including for use in endometrial cancer. Minimally invasive surgery will certainly continue to gain popularity in the future, and maintaining therapeutic effects is a very important challenge. However, laparoscopic and robot-assisted surgeries are limited because the patient cannot be directly touched and it may be difficult to ensure the extrafascial procedure when performing laparoscopic simple total hysterectomy. Therefore, mRH might be more reliable than the extrafascial procedure in laparoscopic surgery for endometrial cancer. It was previously shown that laparoscopic mRH was safe and feasible for the treatment of endometrial cancer [17, 18]. Because laparoscopic surgery for endometrial cancer was only recently introduced, the ideal type of hysterectomy is a topic to be addressed in future studies.

In summary, the present study showed that the rate of local recurrence did not decrease with the selection of a more extensive hysterectomy in surgery for endometrial cancer; thus, it is highly likely that there is little significance in performing mRH instead of SH. However, because the present study had several limitations including its retrospective nature, a prospective study on the rates of local recurrence, PFS, and OS according to the surgical procedure is needed in the future. Furthermore, the indications for laparoscopic surgery and the effectiveness of RH also need to be examined.
